# Gastrointestinal Hyperplasia with Altered Expression of DNA Polymerase β

**DOI:** 10.1371/journal.pone.0006493

**Published:** 2009-08-05

**Authors:** Katsuhiko Yoshizawa, Elena Jelezcova, Ashley R. Brown, Julie F. Foley, Abraham Nyska, Xiangli Cui, Lorne J. Hofseth, Robert M. Maronpot, Samuel H. Wilson, Antonia R. Sepulveda, Robert W. Sobol

**Affiliations:** 1 Department of Pharmacology & Chemical Biology, University of Pittsburgh School of Medicine & University of Pittsburgh Cancer Institute, Hillman Cancer Center, Pittsburgh, Pennsylvania, United States of America; 2 Department of Human Genetics, University of Pittsburgh Graduate School of Public Health, Pittsburgh, Pennsylvania, United States of America; 3 Laboratory of Structural Biology, National Institute of Environmental Health Sciences, Research Triangle Park, North Carolina, United States of America; 4 Cellular and Molecular Pathology Branch, National Institute of Environmental Health Sciences, Research Triangle Park, North Carolina, United States of America; 5 Department of Pathology II, Kansai Medical University, Moriguchi, Osaka, Japan; 6 Department of Pharmaceutical and Biomedical Sciences, South Carolina College of Pharmacy, University of South Carolina, Columbia, South Carolina, United States of America; 7 Department of Pathology and Laboratory Medicine, Hospital of the University of Pennsylvania, Philadelphia, Pennsylvania, United States of America; University of Minnesota, United States of America

## Abstract

**Background:**

Altered expression of DNA polymerase β (Pol β) has been documented in a large percentage of human tumors. However, tumor prevalence or predisposition resulting from Pol β over-expression has not yet been evaluated in a mouse model.

**Methodology/Principal Findings:**

We have recently developed a novel transgenic mouse model that over-expresses Pol β. These mice present with an elevated incidence of spontaneous histologic lesions, including cataracts, hyperplasia of Brunner's gland and mucosal hyperplasia in the duodenum. In addition, osteogenic tumors in mice tails, such as osteoma and osteosarcoma were detected. This is the first report of elevated tumor incidence in a mouse model of Pol β over-expression. These findings prompted an evaluation of human gastrointestinal tumors with regard to Pol β expression. We observed elevated expression of Pol β in stomach adenomas and thyroid follicular carcinomas, but reduced Pol β expression in esophageal adenocarcinomas and squamous carcinomas.

**Conclusions/Significance:**

These data support the hypothesis that balanced and proficient base excision repair protein expression and base excision repair capacity is required for genome stability and protection from hyperplasia and tumor formation.

## Introduction

Increasing evidence is emerging that a large percentage of human tumors have elevated expression of DNA polymerase β (Pol β) [1] and in many cases, mutations within the Pol β coding region results in over-expression of dysfunctional Pol β proteins [2]. High levels of Pol β expression have been demonstrated in several human cancers and tumor cell lines [3]–[6]. Specifically, elevated Pol β expression is observed in esophageal cancer [7], colorectal cancer [8] and pancreatic cancer [9]. Ectopic Pol β expression in human cancer cells is associated with aneuploidy, abnormal localization of centrosome-associated gamma tubulin protein expression during mitosis, increased microsatellite instability [8], [10] and is found to promote tumorigenesis in immunodeficient nude mice [4], [5]. Recently, infection by several viruses associated with elevated cancer incidence, including chronic myelogenous leukemia (CML) [11], human papillomavirus 16 (HPV16) [12] and Epstein-Barr virus (EBV) [13], has been shown to induce the expression of Pol β to elevated levels. Furthermore, approximately 30% of human cancers express mutant or aberrant forms of Pol β proteins [2], [14]–[16], leading to genomic instability and possibly conferring a mutator phenotype to cells [3], [17], [18]. Taken together, current evidence indicates an imbalance in Pol β expression, either increased or decreased, leads to functional deficiency of the base excision repair pathway and promotes genomic instability [3], [17], [18].

As a key enzyme in the base excision repair (BER) pathway, Pol β is essential for the efficient repair of DNA lesions damaged by endogenous and exogenous genotoxins [19]. Once the base lesion is removed and the DNA backbone is hydrolyzed by the concerted action of a lesion-specific DNA glycosylase such as Methyladenine DNA Glycosylase (MPG) and Apurinic/apyrimidinic endonuclease 1 (APE1), the resulting single-nucleotide gap is ‘tailored’ by the 5′dRP lyase activity of Pol β and subsequently, Pol β adds a nucleotide to fill the gap. Repair is then completed by the XRCC1/LigIIIα heterodimer [20]. BER is severely attenuated in the absence of Pol β, leading to an increase in cellular sensitivity to several genotoxins [21]–[26], increased spontaneous and damage-induced mutations and genome rearrangements in knockout (KO) or knockdown (KD) cells [17], [27], [28] and KO (+/−) mice [29]. However, complete KO (−/−) is lethal in mice just after birth [30], [31] preventing detailed analysis of Pol β deficiency beyond embryo development where it is found that Pol β KO neurons die by p53-dependent apoptosis [32] resulting in an increase in mutation frequency in the remaining embryonic tissue [33].

Pol β is involved in many essential protein-protein interactions among the various BER proteins [20], yet some Pol β binding proteins suggest additional functions outside of BER, as evidenced by an interaction with the telomere protein TRF2 [34], the ATM binding protein ATMIN (ASCIZ) [35], [36], the 9-1-1 checkpoint complex [37], the histone acetyltransferase MYST2 [38] or the transcription factor TAF1D (JOSD3, MGC5306) [39]. The functional significance of many of these potential interactions has yet to be revealed. However, Pol β was identified by ChIP analysis as a component of the telomere protein complex [40], a role that is likely related to its interaction with TRF2 [34]. These significant protein-protein interactions and the role of Pol β in BER or other DNA metabolic functions can clearly be impacted by protein expression changes that would disrupt complex formation.

In addition to control via transcription or translation, Pol β is also regulated post-translationally via acetylation [41], methylation [42], [43] and ubiquitylation [44], [45]. These varied modes of Pol β regulation can impact not only Pol β function directly, but changes in expression or in specific post-translational modifications (PTM) can alter function (loss of stability or loss of function due to PTM) or can impact complex formation (loss of protein-protein interactions due to PTM) [20] and lead to repair defects even when Pol β is expressed at high levels.

Transgenic mice with over-expression of the Flag-Pol β transgene were developed here to study the consequences of this effect on imbalanced base excision repair and carcinogenesis. As part of the characterization of this animal model, we report the age-associated histopathological changes present in two-year old Pol β transgenic mice. We find that mice over-expressing Pol β develop Brunner's gland hyperplasia, mucosal hyperplasia in the duodenum and osteogenic tumors in the tail. This was the impetus for an analysis of Pol β expression in relevant human gastrointestinal tumors and the surrounding normal tissue. Paradoxically, we find that whereas stomach adenocarcinoma and thyroid follicular carcinoma present with slightly elevated expression of Pol β, both esophageal squamous carcinoma and esophageal adenocarcinoma show a significant decrease in Pol β expression, compared with surrounding pathologically normal tissue. Overall, these studies support the hypothesis that balanced and proficient BER protein expression and BER capacity is required for genome stability and protection from hyperplasia and tumor formation.

## Results

**Figure 1:**
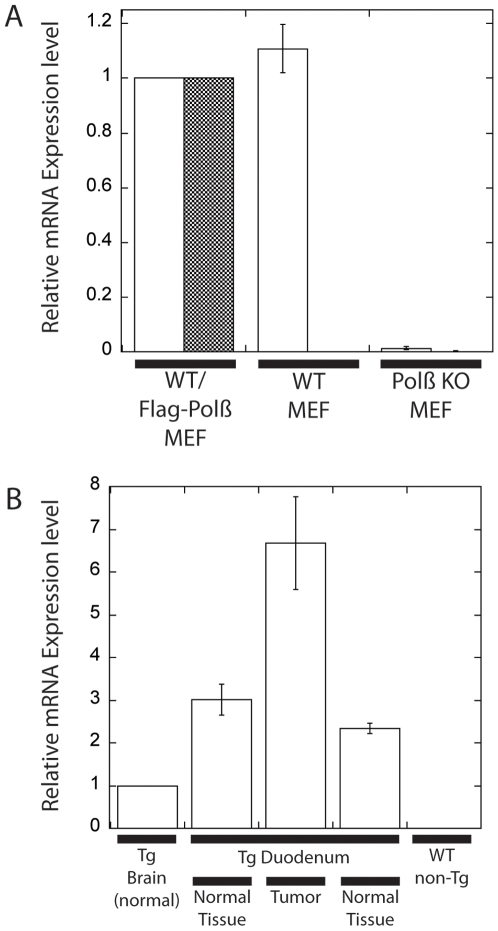
Expression of Flag-Pol β in MEFs and tissues from Pol β Tg mice. (A) Specificity of mouse and human qRT-PCR analysis for Pol β expression: RNA was isolated from WT and Pol β KO MEFs and MEFs expressing the Flag-Pol β transgene, as described in the Methods section. The relative level of expression of both the mouse (open bars) and human (filled bars) Pol β mRNA (normalized to mouse β-actin) was determined using mouse and human specific Taqman assays. Expression across samples was normalized to the expression level in the WT/Flag-Pol β MEF sample. (B) Expression of the human Pol β transgene in mouse tissues and tumors: RNA was isolated from the sample indicated in the plot, as described in the Methods section. The relative level of expression of human Pol β mRNA (open bars; normalized to mouse β-actin) was determined using human specific Taqman assays as in panel A. Expression across samples was normalized to the expression level in the Tg Brain sample.

It is our hypothesis that altered expression of Pol β and the resulting imbalance in BER can predispose to tumor formation. To test this hypothesis, we analyzed transgenic mice that present with elevated expression of Pol β [46]. These Pol β transgenic (Tg) mice express Flag-tagged Pol β (TetOp-Flag-Polβ-tTA) and were described previously [46]. In this present study, the Tg mice were crossed >5 generations to the C57Bl/6 strain. In rare cases, repression of transgene expression has been reported, depending on the transgene, the promoter used for expression and its location [47]–[49]. To verify that expression of the Flag-Pol β transgene was maintained and was not epigenetically silenced during the backcrosses from the original Tg strain to C57BL/6 mice, expression was verified by qRT-PCR of RNA purified from tissue isolated by laser-capture microdissection. The Pol β human transgene used herein has 90.8% sequence identity to the mouse Pol β cDNA (not shown) so we first verified that the Taqman gene expression assays were specific for each mRNA. RNA was isolated from wild-type (WT) mouse embryonic fibroblasts (MEFs), Pol β KO MEFs and WT MEFs that express the Flag-Pol β transgene used in this study. The relative level of expression of either the mouse or human Pol β mRNA was determined for each sample and normalized to the expression of mouse β-actin using the ΔΔC_T_ protocol, as described in the Methods section. As shown in Figure 1A, the expression of both mouse and human Pol β was normalized to the expression level in the transgenic cell line WT/Flag-Pol β. A similar level of expression of mouse Pol β was observed in the WT MEF cell line but no expression of mouse Pol β was detected in the Pol β KO cell line, as expected [21], [22]. Similarly, the expression of the human Pol β transgene was only detected in the WT/Flag-Pol β MEF cells (filled bar, Figure 1A) with no detectable expression in either the WT MEF cells or the Pol β KO MEF cells. This analysis demonstrates the specificity of both the mouse and human Taqman gene expression assays. It should be noted that we used this approach to validate the specificity of a second Taqman gene expression assay for human Pol β (Hs00160263_m1). However, this assay cross-reacted with the mouse mRNA and was not used.

**Figure 2:**
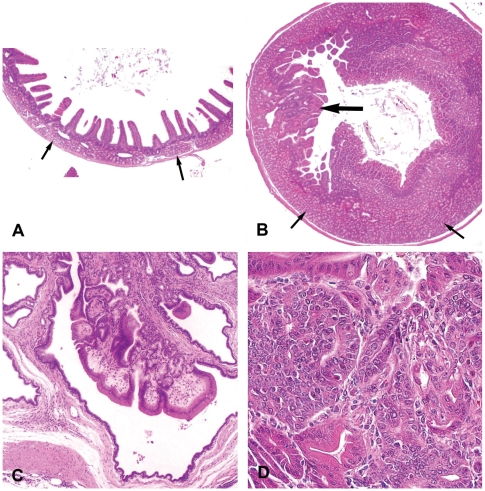
Representative photomicrographs (H & E stain) of duodenal changes in Pol β Tg mice. (A) Normal duodenum. Note normal Brunner's glands (arrows) (magnification×20). (B) Diffuse hyperplasia of Brunner's glands (small arrows) and duodenal crypt epithelium (large arrow). Note markedly increased mucosal thickness due to glandular hyperplasia, compared to panel A (magnification×20). (C) Cystic dilatation of mucosal crypts and Brunner's glands with displacement of cystic glands into the tunica muscularis (magnification×200). (D) Focal proliferation of dysplastic glands in a mouse diagnosed with duodenal adenoma (magnification×400).

Using the validated gene expression assays and the ΔΔC_T_ protocol as described above and in the Methods section, we next determined if the human Flag-Pol β transgene was expressed in the cells of the tissues of interest and most importantly, in the tumors. Using laser-capture microdissection, we isolated and purified RNA from the brain (not shown) and the normal and tumor samples described in Figure 2, as well as from cells from a non-transgenic (non-Tg) mouse. Previous studies (not shown) suggested that the expression of the Flag-Pol β transgene in the brain was low but detectable [46] and so the relative quantitation of Flag-Pol β expression was normalized to the level of expression in the brain (Figure 1B). As shown, the level of expression in normal duodenum tissue was slightly elevated as compared to the brain, similar to that observed in our previous analysis of this Tg mouse [46]. However, expression in the hyperplastic duodenum was approximately 7-fold higher than the brain and 2- to 3-fold higher than the normal duodenum tissue. These studies therefore confirm that the Flag-Pol β transgene is expressed at elevated levels depending on the tissue and most importantly, the transgene expression appears to be further elevated in the tumors (Figure 1B). Further, these results suggest that the expression of the Flag-Pol β transgene is elevated in these mice similar to that reported earlier [46].

We therefore evaluated the spectrum of pathological lesions in a cohort of our Pol β Tg mice at 2 years of age. Macroscopic lesions are summarized in Table 1 and non-neoplastic and neoplastic lesions are summarized in Tables 2 through [Table pone-0006493-t003] 4. Morphological characteristics of some of the main lesions are as follows in the sections below.

**Table 1:** Incidence of macroscopic findings observed in DNA polymerase β Tg mice.

Organ	Macroscopic Finding	Frequency (%)*	
		Male (n = 15)	Female (n = 21)
Abdominal cavity	Fluid	13.3	19.0
	Mass/Nodule	6.7	0.0
Heart	Enlarged	13.3	0.0
	Soft	0.0	4.8
Salivary gland	Atrophy	0.0	28.6
	Mass/Nodule	0.0	9.5
Liver	Cysts	6.7	28.6
	Enlarged	0.0	9.5
	Mottled	20.0	9.5
	Mass/Nodule	20.0	9.5
Duodenum	Thickened, red	0.0	14.3
	Mass/Nodule	13.3	23.8
Jejunum	Mass	6.7	4.8
Ileum	Mass	0.0	4.8
Colon	Mass	6.7	0.0
Adrenal	Enlarged	6.7	4.8
Pituitary gland	Dark, cystic	0.0	4.8
Spleen	Enlarged	6.7	38.0
	White Foci	6.7	0.0
	Small/Atrophy	6.7	4.8
	Mass/Nodule	6.7	4.8
Thymus	Enlarged	0.0	4.8
Mesenteric Lymph Node	Enlarged	40.0	48
	Mass/Nodule	0.0	4.8
Other Lymph Node	Enlarged	20.0	38.1
Testis	Dark red	6.7	–
Seminal vesicle	Black, cystic	6.7	–
	Enlarged	20.0	–
Prostate	Enlarged	6.7	–
Ovary	Dark red	–	19.0
Uterus	Dilated	–	4.8
Lung	Pale	6.7	0.0
Ear	Ulcer	0.0	4.8
Eye	Lens, white	100	100
Tail	Mass/Nodule	33.3	14.3

* Frequency defined as the number of animals with the lesion divided by the number of animals with the tissue examined macroscopically, multiplied by 100.

**Table 2:** Incidence of non-neoplastic lesions observed in DNA polymerase β Tg mice.

Organ	Macroscopic Finding	Frequency (%)*	
		Male (n = 15)	Female (n = 21)
Heart	Polyarteritis	6.7	0.0
Salivary gland	Mononuclear cell, infiltration	93.3	45.0
Duodenum	Erosion	0.0	9.5
	Ectopic pancreas	0.0	4.8
Liver	Altered cell foci	6.7	4.8
	Biliary cyst	6.7	14.3
	Chronic active hepatitis	13.3	0.0
	Erythrophagocytosis	0.0	4.8
	Extramedullary hematopoiesis	6.7	14.3
	Hemorrhage	20.0	4.8
	Hyaline bodies, cytoplasm	6.7	0.0
	Microgranulation	0.0	4.8
	Mononuclear cell, infiltration	53.3	47.6
	Necrosis	13.3	14.3
	Pigmentation	0.0	4.8
	Subcapsular infiltration	0.0	4.8
Gallbladder	Hyaline inclusion	27.3	5.0
Pancreas	Mononuclear cell, infiltration	0.0	19.0
Adrenal gland	Pigmentation	33.3	95.2
Mesenteric Lymph Node	Erythrophagocytosis	0.0	8.3
Other Lymph Node	Plasmacytosis	6.7	0.0
Spleen	Congestion	0.0	4.8
	Extramedullary hematopoiesis	73.3	75.0
Mammary gland	Lactation	–	5.6
Testis	Sperm granuloma	14.3	–
	Hemorrhage	7.1	–
	Tubular, atrophy	7.1	–
Epididymis	Hypospermia	7.7	–
Prostate	Mononuclear cell, infiltration	6.7	
	Polyarteritis	6.7	
Seminal vesicle	Hemorrhage	6.7	

* Frequency defined as the number of animals with the lesion divided by the number of animals with the tissue examined histopathologically, multiplied by 100. Abnormal non-neoplastic changes were not detected in thyroid, parathyroid, thymus, stomach, jejunum, ileum, cecum, colon and pituitary.

**Table 3 pone-0006493-t003:** Incidence of non-neoplastic lesions observed in DNA polymerase β Tg mice.

Organ	Macroscopic Finding	Frequency (%)*	
		Male (n = 15)	Female (n = 21)
Ovary	Atrophy	–	94.4
	Cyst	–	60.0
	Thrombus	–	5.6
Vagina	Erosion	–	50.0
Lung	Mononuclear cell, infiltration	60.0	57.1
Eye	Cataract	100.0	100.0
Kidney	Basophilic tubules	86.7	80.0
	Glomerular, hyalinization	100.0	85.0
	Glomerular, hypercellularity	20.0	20.0
	Mononuclear cell, infiltration	80.0	85.0
	Tubular, vacuolization	93.3	0.0
	Hyaline droplet	0.0	15.0
	Protein cast	0.0	10.0
	Pelvic, dilatation	0.0	5.0
Skin	Ulcer	0.0	5.0
	Crust	0.0	5.0
	Mononuclear cell, infiltration	0.0	10.0
Brain	Mineralization	53.3	15.8
	Hemosiderin deposition	6.7	0.0
	Lateral ventricle, dilatation	13.3	0.0
	Mononuclear cell, infiltration	0.0	5.3
	Polyarteritis	6.7	0.0
Urinary bladder	Mononuclear cell, infiltration	8.3	38.1
Tail	Chondro-osseous metaplasia	–	33.3

* Frequency defined as the number of animals with the lesion divided by the number of animals with the tissue examined histopathologically, multiplied by 100. Abnormal non-neoplastic changes were not detected in thyroid, parathyroid, thymus, stomach, jejunum, ileum, cecum, colon and pituitary.

**Table 4:** Incidence of non-neoplastic & neoplastic proliferative lesions in DNA polymerase β Tg mice.

Organ	Lesion	Frequency (%)*		Organ	Lesion	Frequency (%)*	
		Male	Female			Male	Female
Salivary gland	Lymphoid hyperplasia	0.0	10.0	Other Lymph Node	Lymphoid hyperplasia	5.6	3.8
	Malignant lymphoma	0.0	15.0		Malignant lymphoma	11.1	15.4
Forestomach	Squamous hyperplasia	0.0	5.0		Histiocytic sarcoma	0.0	30.8
Duodenum	Bruner's gland/Mucosa Hyperplasia,	26.7	47.6	Spleen	Lymphoid hyperplasia	13.3	25.0
					Malignant lymphoma	6.7	5.0
					Histiocytic sarcoma	6.7	5.0
	Adenoma	0.0	4.8	Thymus	Malignant lymphoma	0.0	50.0
	Malignant lymphoma	6.7	4.8	Skin	Lymphoid hyperplasia	6.7	5.0
Jejunum	Malignant lymphoma	6.7	10.0		Squamous, hyperplasia	0.0	5.0
Liver	Adenoma	13.3	0.0	Ovary	Malignant lymphoma	–	5.6
	Histiocytic sarcoma	13.3	14.3		Histiocytic sarcoma	–	5.6
Adrenal gland	Cortical hyperplasia	16.7	9.5	Uterus	Endometrial, hyperplasia	–	31.6
	Cortical spindle cell hyperplasia	0.0	90.5	Lung	Lymphoid hyperplasia	6.7	9.5
Thyroid	Follicular cell, hyperplasia	0.0	18.8		Malignant lymphoma	0.0	9.5
	Follicular cell adenoma	0.0	6.3		Histiocytic sarcoma	6.7	4.8
Pituitary gland	Hyperplasia	0.0	25.0		Bronchiolar-alveolar adenoma	20.0	0.0
Mesenteric Lymph Node	Lymphoid hyperplasia	9.0	0.0	Kidney	Malignant lymphoma	0.0	5.0
	Malignant lymphoma	63.6	58.3	Tail	Osteoma	60.0	0.0
	Histiocytic sarcoma	9.0	25.0		Osteosarcoma	20.0	33.3

* Frequency defined as the number of animals with the lesion divided by the number of animals with the tissue examined histopathologically, multiplied by 100. Data derived from 15 males and 21 females except where noted. No neoplastic changes were detected in gallbladder, parathyroid, thymus, ileum, cecum, colon, pancreas, brain, eye, urinary bladder, testis, epididymis, prostate, seminal vesicle, oviduct, vagina, and mammary gland.

### Proliferative lesions of the duodenum

Hyperplasia of Brunner's glands and hyperplasia of the duodenal crypt epithelium resulting in markedly thickened duodenal mucosa was detected in four of 15 male mice (26.7%) and ten of 21 female mice (47.6%) (compare Figure 2A and 2B). Proliferative lesions, especially around Vater's papilla, were detected with high incidence. Such changes were characterized by diffuse epithelial hyperplasia, altered epithelial differentiation, eosinophilic cytoplasmic inclusions, and herniation/diverticulation (Figure 2C) of the epithelium into the tunica muscularis and serosa. Adenoma of relatively small size, was diagnosed in one of 21 female mice (Figure 2D). Proliferative lesions were often associated with chronic granulomatous inflammation and are similar to duodenal plaques (avillous hyperplasia, duodenum polyp) derived from the crypts of Lieberkuhn [50]. Most of these lesions are regarded as benign hyperplastic lesions, but some may develop dysplasia, a pre-malignant neoplastic lesion.

### Osteogenic tumors in tail

**Figure 3:**
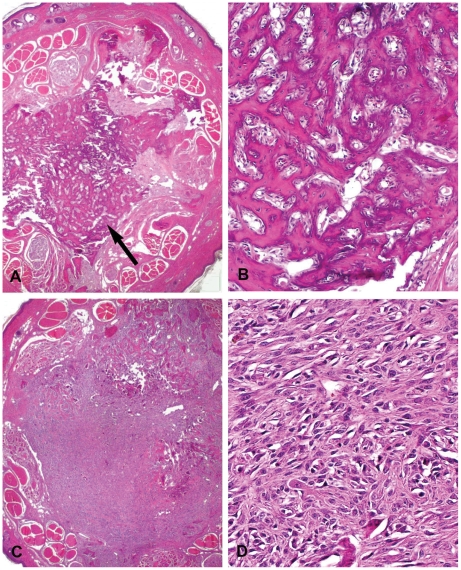
Osteogenic tumors of tails in Pol β Tg mice (H & E stain). (A) Osteoma (arrow) in the central area of tail (magnification×20, Decalcified). (B) Higher magnification of panel A. Irregular trabecular formation by spindle tumor cells (magnification×200). (C) Osteosarcoma. The tumor occupied almost the entire subcutaneous area of the tail (magnification×20). (D) Spindle tumor cells resemble fibroblastic mesenchymal cells and proliferated with production of a small amount of osteoid deposition (magnification×400).

Macroscopically, masses or nodules in the tail were detected in 33.3% (5/15) of male and 14.3% (3/21) of female mice. Histopathologically, chondro-osseous metaplasia was detected in one of 3 female mice; osteoma was detected in three of 5 male mice (Figure 3A and 3B); and osteosarcoma was detected in one of 5 male and one of 3 female mice (Figure 3C and 3D). Osteosarcoma was characterized by proliferating spindle tumor cells, associated with presence of irregular, infiltrating trabecular bone. In this study, osteogenic tumors were not detected in any other site besides the tail.

### Mature Cataract

**Figure 4:**
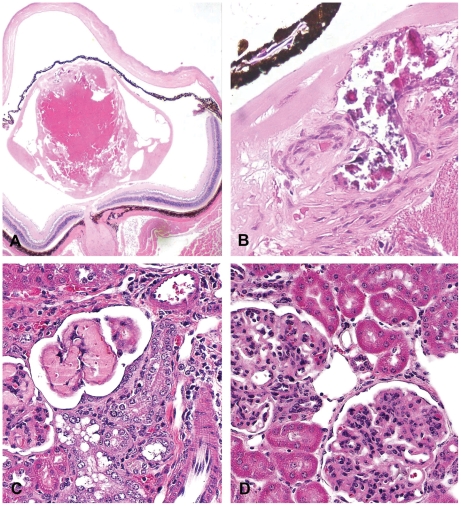
Representative photomicrographs (H & E stain) of lenticular and glomerular damages in Pol β Tg mice. (A) Mature cataract characterized by degeneration/necrosis (liquefaction) and vacuolar formation in lens fibers (magnification×20). (B) High-magnification of figure a. Irregular proliferation of spindle lens fiber cells without production of normal lens fibers; necrosis and calcification are present (magnification×400). (C) Glomerular hyalinization with basement membrane thickening of Bowman's capsule, basophilic tubules, and tubular vacuolation in the renal cortex (magnification×400). (D) Glomerular hypercellularity with basement membrane thickening (magnification×400).

Mature cataracts were detected in all mice examined (male; 13/13, female; 21/21), essentially as we described previously [46]. Histopathologically, the lens changes were characterized by degeneration/necrosis (liquefaction), vacuole formation in most of the lens fibers, and irregular proliferation of lens fiber spindle cells, followed by calcification (Figure 4A and 4B). Moreover, lens epithelia with bizarre nuclei and single cell necrosis in the proliferative fiber cells were seen. In all cases, lenticular lesions were diagnosed as a mature type, a final stage of cataract formation.

### Renal lesions

A spectrum of renal lesions was detected in almost all animals with glomerular changes, basophilic tubules, and mononuclear cell infiltration occurring in both sexes. Glomerular hyalinization was detected in almost all mice (male; 15/15, female; 17/20) with basement membrane thickening (Figure 4C and 4D). In mild cases, eosinophilic material in glomerular basement membranes was seen, while densely eosinophilic and amorphous deposits in glomeruli were seen in severe cases. In addition, glomerular hypercellularity, consistent with proliferation of mesangial cells, was present in 3 of 15 male and 4 of 20 female mice. Moreover, basophilic tubules observed in the cortex of 13 of 15 male and 16 of 20 female mice. These lesions are representative of glomerulonephritis.

### Expression of Pol β in human tumors and surrounding normal tissue

**Figure 5:**
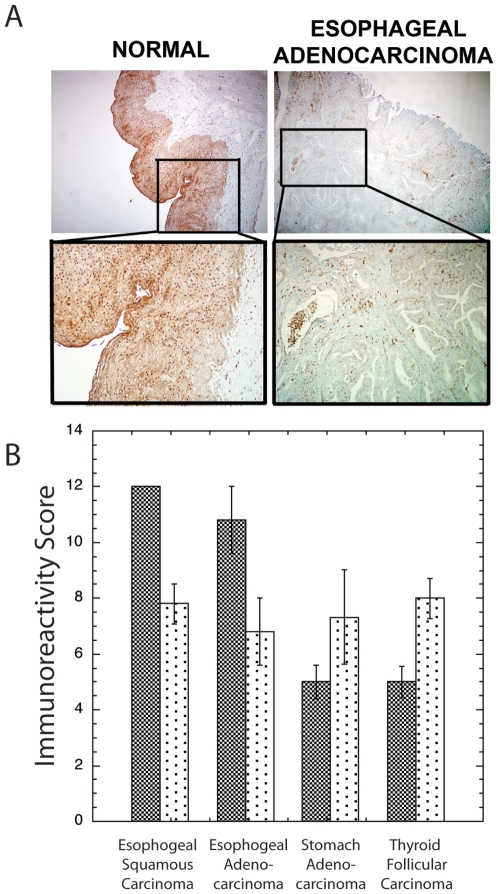
Decreased expression of Pol β in human esophageal adenocarcinoma. (A) Photomicrograph of sections of esophageal adenocarcinoma and esophageal squamous mucosa stained for Pol β expression by immunohistochemistry. Top images reflect magnification×40 and the inserts depict magnification×100. (B) Bar graph representing relative expression level of Pol β in various tumors (stippled, open bars) and pathologically normal (grey bars) epithelial tissues. Immunoreactivity Score is the average of 5 different tumor samples each evaluated in two independent analyses.

To extend observations in mice, we examined Pol β expression in human gastrointestinal cancers and in surrounding normal epithelial tissues (Figure 5A). Because cancers are more likely to arise in epithelial tissues, we separated scores in epithelial and stromal (subepithelial) areas. There was no significant difference in Pol β immunoreactivity scores between stromal areas in cancerous vs. surrounding non-cancerous tissues. In epithelial tissues, we observed (Figure 5B) elevated expression of Pol β in stomach adenocarcinomas compared with surrounding normal gastric mucosal tissue, although this difference was not significant (p = 0.19). Compared with paired normal surrounding tissues, however, there was a significant increase in Pol β in thyroid follicular carcinomas (p = 0.02), but significantly reduced Pol β expression in esophageal adenocarcinomas (p = 0.04) and squamous carcinomas (p = 0.005).

## Discussion

BER proteins require a finely tuned balance of expression to ensure complete repair of many mutagenic or genome destabilizing base lesions [24], [46], [51]–[54]. Altered expression or mutations in BER proteins such as Pol β that impact function or protein-protein interactions can predispose to sensitivity to genotoxins [22], an increase in genome alterations [17], mutations [27] and tumor formation [55]. In total, cellular, epidemiological and pathological analyses suggested a correlation between several human cancers and Pol β mutations and/or expression changes.

In cell-based studies, alteration in expression of Pol β impacts BER capacity and manifests as a genome destabilizing phenotype, consistent with the observation that greater than 30% of human tumors have elevated expression of Pol β [1] and in a separate study it was revealed that greater than 30% of human tumors express mutant forms of Pol β [2]. Only a few animal models with altered Pol β expression have been characterized to study how alterations in Pol β expression might impact tumor formation in the whole animal. Mice with a partial deficiency in Pol β expression (Pol β heterozygote mice) have an elevated mutant frequency in male germ cells [27] and a small increase in the incidence of lymphoid hyperplasia and adenocarcinoma [56]. Mice expressing a truncated form of Pol β (polbetaΔ) in mammary glands showed an elevated incidence of tumor formation [55]. This mutant of Pol β is a splice-variant missing amino acids 208–236 [57] however, this variant is not cancer specific [58]. A second Pol β Tg mouse was developed but this model only expresses Pol β in the thymus and elevated tumor formation was not observed [59].

The development of the Pol β transgenic mice used in this study and the Pol β expression pattern was described previously [46]. The Pol β Tg mice were backcrossed onto a C57BL background, a strain widely used for development of transgenic mice and gene-targeting experiments [60]. The majority of non-neoplastic and neoplastic lesions observed in the Pol β Tg mice were considered spontaneous and age-related as previously reported in C57BL and other mouse strains [50], [60]–[63]. The more commonly occurring spontaneous lesions in C57BL/6 (e.g., lymphoma and histiocytic sarcoma) were not increased in the Pol β Tg mice. Lesions unique to the Pol β Tg mice were found in the duodenum, tail, eye, and kidney. The increased incidence (100%) of lesions in the eye (cataract) was described earlier [46].

The 39% incidence of proliferative duodenal lesions in the Pol β Tg mice is considerably higher than previously reported incidences of 4% [63] and 21% [64] in aged C57BL mice. Other spontaneous plaquelike lesions or polyposis in the pyloric area of the glandular stomach have been observed in C57BL/Ncr×129/SvTer (B6,129) mice, 129/SvTer mice, Ahr-null mice, TGF b-1 heterozygous mice, Smad4 heterozygous mice, CYP1A2-null mice, and B6C3F1 mice [65]–[70]. Moreover, C57BL mice have been reported to be very susceptible to duodenal neoplasia following some carcinogen treatments [71]. A majority of the non-neoplastic lesions are considered spontaneous age-related and have been reported in C57BL/6 and other mouse strains. The incidence of the non-neoplastic lesions was not increased in the DNA Polymerase β over-expressing Tg mice. In particular, mononuclear cell infiltration in all of the organs (mainly lymphocytes), ovarian cystic lesions and atrophy, and liver lesions are very common lesions in aged C57BL/6 mice. As well, there was no gender differences. Therefore, Pol β Tg mice might also be suitable models for duodenal carcinogenicity following treatment with appropriate carcinogens.

Duodenal epithelial tumors that develop from the intestinal type or the pancreaticobiliary type mucosa of Vater's papilla [72], [73] are relatively rare tumors in humans; the incidence rate of adenoma is 0.04–0.62% and that of carcinoma is 0.2% in postmortem or autopsy studies [74]. Molecular alterations in these duodenal lesions are similar to those of colorectal tumors and include K-ras mutation and the overexpression of p53, p21/Waf1, p16, and/or APC [72], [73], [75]. Mice which carry a mutation in the Apc gene have multiple neoplastic lesions in duodenum (42%), jejunum (38%), stomach (25%), ileum (15%), and colon (8%) [76]. It is not known if altered expression of the above-mentioned genes might be related to the pathogenesis of duodenal lesions in Pol β Tg mice. Recently, it was reported that APC directly inhibits BER [77]. It is therefore possible that APC functions to regulate BER, suggesting that Pol β over-expression may lead to a similar phenotype as APC deficiency.

Spontaneous occurrence of osteogenic tumors is extremely rare in rats and mice, and there are only a few reports published with incidences of 0.1 to 2% in mice and 0 to 4% in rats [50], [78]–[83]. Therefore, the incidence of osteogenic tumors in Pol β Tg mice is considerably higher than incidences cited in the literature. While the vertebral column including caudal vertebrae (tail) has previously been noted as a common site of osteogenic tumors in mice [81], most reports do not detail the specific sites of osteogenic bone tumors.

Glomerulonephritis and related renal lesions are common findings in C57BL/6 mice [60]. However, it is not clear whether the incidence of glomerulonephritis in the Pol β model is higher that that in C57BL/6 mice due to lack of available data in two-year old C57BL/6 mice.

A higher level of Pol β expression has been found in human intestinal adenocarcinoma than in other organs tumors [3], [84]. In this study, we observed a variable outcome in that ESC and EA presented with decreased expression of Pol β whereas SA and TFC showed elevated Pol β expression. In all cases, the efficiency of the DNA repair system (Pol β) might be compromised due to altered PTM or complex formation [84]. Unfortunately, there have been no reports about the relationship between Pol β expression and osteogenic tumors in humans and animals. Bergoglio and colleagues reported that tumor induction could not be seen in a Pol β Tg mouse model with thymus-specific Pol β transgene expression, suggesting that Pol β over-expression is not sufficient to initiate tumorigenesis *in vivo* [59]. In our Pol β Tg mice, over-expression of Pol β in most systemic tissues was confirmed and the degree of over-expression of Pol β in small intestine was shown to be similar to that in lens [46]. Therefore, we believe that the relationship between lesion pathogenesis and over-expression of Pol β might reflect organ specificity.

The relationship between Pol β over-expression and human carcinogenesis remains to be elucidated. Previous reviews have reported that greater than 30% of human tumors have elevated expression of Pol β [1] or express dysfunctional Pol β proteins [2]. Additional research is needed to analyze the relationship between Pol β over-expression and intestinal carcinogenesis and the understanding of the potential extrapolations from our model to humans due to over-expression of Pol β. This mouse model might be a useful tool for cancer chemotherapy as well as evaluating the environmental and genetic factors that cooperate with Pol β expression variation to impact hyperplasia and tumor formation.

## Materials and Methods

### Animals

DNA polymerase Pol β transgenic mice were described previously [46]. Genotyping was as described [46]. These mice (fifteen male and twenty-one female), express Flag-tagged Pol β (TetOp-Flag_polβ-tTA) and were developed in B6SJL-hybrid females and then back-crossed onto C57BL/6 mice [46]. The Pol β expression pattern and the level of over-expression was described previously [46]. These mice over-express Pol β in almost all organs, including stomach and small intestine [46]. This mouse strain is available from the NIH-sponsored Mutant Mouse Regional Resource Centers (MMRRC) (Strain name: B6.Cg-Tg(TetOp-Polb/tTA)2Sbl/Mmmh, Stock number: 000356-MU). Details for this strain are available at http://www.mmrrc.org/strains/356/0356.html. All breeding was at NIH using IACUC and ALAAS approved protocols for the duration of the study. The transgenic mice used in the study were crossed to C57BL/6 (Taconic) for >5 generations, as in the previous study [46]. Each Tg animal is considered a heterozygous Tg mouse in that breeding was only performed using either a transgenic male and C57BL/6 female or a C57BL/6 male and transgenic female. Transgenic mice were never inter-bred. Mice were housed in solid-bottom polycarbonate cages. Filtered room air underwent at least 10 changes per hour. The animal room was maintained at 22 +/− 2°C with 50 +/− 15% relative humidity and a 12-hour light-dark cycle. Irradiated NTP-2000 pelleted feed (Zeigler Bros., Inc., Gardner, PA) and water were available *ad libitum*. Animal handling and husbandry were conducted in accordance with NIH guidelines [85].

### Pathology

Necropsies were performed on all mice after euthanization at the age of 24 months. Euthanasia was by asphyxiation with carbon dioxide and mice were necropsied within 5 min of death. At necropsy, all tissues including masses and macroscopical abnormalities were removed and fixed in 10% neutral buffered formalin. After fixation, the following tissues were trimmed, dehydrated, cleared, and paraffin-embedded: liver, gallbladder, lung, thyroid gland, parathyroid gland, salivary gland, spleen, heart, kidney, stomach, duodenum, jejunum, ileum, cecum, colon, pancreas, mesenteric lymph node, skin, mammary gland, brain, eye, urinary bladder, testis, epididymis, prostate, seminal vesicle, ovary, uterus, vagina, adrenal gland, and pituitary gland. Other lymph nodes (mediastinal, pancreatic, cervical, and/or perirenal) were examined when macroscopical lesions were detected. In addition, for animals that had macroscopical tail lesions, samples were decalcified prior to routine processing. Five-micron thick sections were mounted onto glass slides, stained with hematoxylin and eosin (H&E), and examined microscopically. The severity of non-neoplastic lesions was graded on a four-point scale of 1 = minimal, 2 = mild, 3 = moderate, and 4 = marked. Histopathology evaluation was performed by two pathologists (KY and AN). Previously published histopathological terminology and diagnostic criteria were used [50], [60], [63], [64].

### Laser-Capture Microdisection of Paraffin-Embedded tissue and tumor samples

RNA was isolated from laser capture micro-dissected Formalin-Fixed Paraffin-Embedded (FFPE) tissue and tumor samples using the Cellcut™ instrument (Molecular Machines and Industries, Haslett, MI). Two sections (8 µm) were placed on five polyethylene terephthalate (PET) foil slides. A detailed protocol for staining and the LM process is available on the NIEHS Laser Microdissection Core Facility web site [86], [87]. Using the PureLink™ FFPE RNA Isolation Kit (Invitrogen Life Technologies, Carlsbad, CA.), the LM samples and whole section controls were lysed and RNA isolated on the same day.

### Quantitative RT-PCR Analysis

Expression of mouse and human Pol β mRNA was measured by quantitative RT-PCR using an Applied Biosystems StepOnePlus system. Briefly, 80,000 MEF cells (WT, WT expressing the Flag-Pol β transgene and Pol β KO) were lysed and reverse transcribed using the Applied Biosystems Taqman® Gene Expression Cells-to-CT™ Kit. Each sample was analyzed in triplicate and the results shown are an average of all three analyses. Analysis of mRNA expression was conducted as per the manufacturer (ΔΔC_T_ method) using Applied Biosystems TaqMan® Gene Expression Assays (human POL β: part #4331182, Hs01099715_m1; mouse Pol β: part #4331182, Mm00448234_m1) and normalized to the expression of mouse β-actin (part #4352933E).

For the analysis of the Tg mouse tissue, RNA was extracted as described above and cDNA was synthesized from 30 ng of RNA using the Applied Biosystems High Capacity cDNA Reverse Transcription Kit (part #4375575). The cDNA was pre-amplified for 10 cycles using the TaqMan® PreAmp Master Mix (part # 4391128) and diluted 1∶5. The pre-amplified cDNA was next analyzed using the Applied Biosystems TaqMan® Gene Expression Assays (human POL β: part #4331182, Hs01099715_m1; mouse Pol β: part #4331182, Mm00448234_m1) and normalized to the expression of mouse β-actin. Expression analysis was determined using the ΔΔC_T_ protocol as per the manufacturer to determine the relative quantitation of Flag-Pol β expression, as compared to the mouse β-actin among all samples. From the tissue samples, expression was normalized to the level of expression in the brain of Tg mice.

### Immunohistochemistry for Pol β in human epithelial tumors

Tissues samples [Esophageal Squamous Carcinoma (ESC), Esophageal Adenocarcinoma (EA), Thyroid Follicular Carcinoma (TFC) and Stomach Adenocarcinoma (SA) and surrounding normal tissue] were obtained through the Tissue and Research pathology Services (TARPS), University of Pittsburgh Cancer Institute. Five cases were examined for each of the types of carcinoma. Five-micron thick sections of paraffin-embedded tissue on glass slides were either stained with hematoxylin and eosin (H&E) or analyzed for Pol β expression by immunohistochemistry. Labeling was performed on formalin-fixed, paraffin-embedded tissues by incubation with antibodies against Pol β (Abcam, polyclonal, cat#AB53059, diluted 1 in 5000). To ensure even staining and reproducible results, sections were incubated by slow rocking overnight in primary antibody (4°C) using the Antibody Amplifier™(ProHisto, LLC, Columbia, SC). Following incubation with primary antibody, sections were processed with a rabbit polyclonal EnVision+System-HRP kit (DakoCytomation, Carpinteria, CA) according to the kit protocols. The chromogen was diaminobenzidene and sections were counter stained with 1% methyl green. The negative controls were tissues from Pol β knockout mice, which were negative for staining. Immunohistochemistry was quantified by two independent investigators in a blind fashion as previously described [88]. Cases with a disagreement of both investigators on the immunoreactive score were discussed using a multiheaded microscope until consensus was achieved. A score was calculated based of the percentage of positive tumor cells (<10% = 1; 11–50% = 2; 51–80% = 3; >80% = 4) multiplied by the staining intensity (negative = 0; weak = 1; moderate = 2; strong = 3). For the immunoreactive score (IRS) the scores for the percentage of positive cells and the staining intensity were multiplied, resulting in a value between 0 and 12. For immunohistochemical quantification, mean differences between groups were compared by one-way analysis of variance with Scheffe multiple comparison tests. The P-value chosen for significance in this study was 0.05.

## References

[1] Albertella MR, Lau A, O'Connor MJ (2005). The overexpression of specialized DNA polymerases in cancer.. DNA Repair (Amst).

[2] Starcevic D, Dalal S, Sweasy JB (2004). Is there a link between DNA polymerase beta and cancer?. Cell Cycle.

[3] Srivastava DK, Husain I, Arteaga CL, Wilson SH (1999). DNA polymerase β expression differences in selected human tumors and cell lines.. Carcinogenesis.

[4] Bergoglio V, Canitrot Y, Hogarth L, Minto L, Howell SB (2001). Enhanced expression and activity of DNA polymerase β in human ovarian tumor cells: impact on sensitivity towards antitumor agents.. Oncogene.

[5] Bergoglio V, Pillaire MJ, Lacroix-Triki M, Raynaud-Messina B, Canitrot Y (2002). Deregulated DNA polymerase β induces chromosome instability and tumorigenesis.. Cancer Research.

[6] Servant L, Bieth A, Hayakawa H, Cazaux C, Hoffmann JS (2002). Involvement of DNA polymerase β in DNA replication and mutagenic consequences.. Journal of Molecular Biology.

[7] Dong ZM, Zheng NG, Wu JL, Li SK, Wang YL (2006). Difference in expression level and localization of DNA polymerase beta among human esophageal cancer focus, adjacent and corresponding normal tissues.. Dis Esophagus.

[8] Yu J, Mallon MA, Zhang W, Freimuth RR, Marsh S (2006). DNA repair pathway profiling and microsatellite instability in colorectal cancer.. Clin Cancer Res.

[9] Fan R, Kumaravel TS, Jalali F, Marrano P, Squire JA (2004). Defective DNA strand break repair after DNA damage in prostate cancer cells: implications for genetic instability and prostate cancer progression.. Cancer Res.

[10] Yamada NA, Farber RA (2002). Induction of a low level of microsatellite instability by overexpression of DNA polymerase Beta.. Cancer Res.

[11] Canitrot Y, Laurent G, Astarie-Dequeker C, Bordier C, Cazaux C (2006). Enhanced expression and activity of DNA polymerase beta in chronic myelogenous leukemia.. Anticancer Res.

[12] Liu SN, Bai WY, Frye RM, Hou L, Zhang B (2008). Specific up-regulation of DNA polymerase by human papillomavirus 16.. Chin Med Sci J.

[13] Faumont N, Le Clorennec C, Teira P, Goormachtigh G, Coll J (2009). Regulation of DNA polymerase beta by the LMP1 oncoprotein of EBV through the nuclear factor-kappaB pathway.. Cancer Res.

[14] Michiels S, Laplanche A, Boulet T, Dessen P, Guillonneau B (2009). Genetic polymorphisms in 85 DNA repair genes and bladder cancer risk.. Carcinogenesis.

[15] Li D, Suzuki H, Liu B, Morris J, Liu J (2009). DNA repair gene polymorphisms and risk of pancreatic cancer.. Clin Cancer Res.

[16] Li D, Li Y, Jiao L, Chang DZ, Beinart G (2007). Effects of base excision repair gene polymorphisms on pancreatic cancer survival.. Int J Cancer.

[17] Sobol RW, Kartalou M, Almeida KH, Joyce DF, Engelward BP (2003). Base Excision Repair Intermediates Induce p53-independent Cytotoxic and Genotoxic Responses.. Journal of Biological Chemistry.

[18] Sobol RW, Watson DE, Nakamura J, Yakes FM, Hou E (2002). Mutations associated with base excision repair deficiency and methylation-induced genotoxic stress.. Proceedings of the National Academy of Science.

[19] Wood RD, Mitchell M, Sgouros J, Lindahl T (2001). Human DNA repair genes.. Science.

[20] Almeida KH, Sobol RW (2007). A unified view of base excision repair: lesion-dependent protein complexes regulated by post-translational modification.. DNA Repair.

[21] Trivedi RN, Almeida KH, Fornsaglio JL, Schamus S, Sobol RW (2005). The Role of Base Excision Repair in the Sensitivity and Resistance to Temozolomide Mediated Cell Death.. Cancer Research.

[22] Sobol RW, Horton JK, Kuhn R, Gu H, Singhal RK (1996). Requirement of mammalian DNA polymerase-β in base-excision repair.. Nature.

[23] Sobol RW, Prasad R, Evenski A, Baker A, Yang XP (2000). The lyase activity of the DNA repair protein β-polymerase protects from DNA-damage-induced cytotoxicity.. Nature.

[24] Trivedi RN, Wang XH, Jelezcova E, Goellner EM, Tang J (2008). Human methyl purine DNA glycosylase and DNA polymerase β expression collectively predict sensitivity to temozolomide.. Molecular Pharmacology.

[25] Horton JK, Baker A, Berg BJ, Sobol RW, Wilson SH (2002). Involvement of DNA polymerase β in protection against the cytotoxicity of oxidative DNA damage.. DNA Repair (Amst).

[26] Ochs K, Lips J, Profittlich S, Kaina B (2002). Deficiency in DNA polymerase β provokes replication-dependent apoptosis via DNA breakage, Bcl-2 decline and caspase-3/9 activation.. Cancer Res.

[27] Allen D, Herbert DC, McMahan CA, Rotrekl V, Sobol RW (2008). Mutagenesis is elevated in male germ cells obtained from DNA polymerase-beta heterozygous mice.. Biol Reprod.

[28] Pascucci B, Russo MT, Crescenzi M, Bignami M, Dogliotti E (2005). The accumulation of MMS-induced single strand breaks in G1 phase is recombinogenic in DNA polymerase beta defective mammalian cells.. Nucleic Acids Res.

[29] Cabelof DC, Guo Z, Raffoul JJ, Sobol RW, Wilson SH (2003). Base excision repair deficiency caused by polymerase β haploinsufficiency: accelerated DNA damage and increased mutational response to carcinogens.. Cancer Research.

[30] Gu H, Marth JD, Orban PC, Mossmann H, Rajewsky K (1994). Deletion of a DNA polymerase β gene segment in T cells using cell type-specific gene targeting.. Science.

[31] Sugo N, Aratani Y, Nagashima Y, Kubota Y, Koyama H (2000). Neonatal lethality with abnormal neurogenesis in mice deficient in DNA polymerase β.. EMBO Journal.

[32] Sugo N, Niimi N, Aratani Y, Takiguchi-Hayashi K, Koyama H (2004). p53 Deficiency rescues neuronal apoptosis but not differentiation in DNA polymerase β-deficient mice.. Molecular and Cellular Biology.

[33] Niimi N, Sugo N, Aratani Y, Gondo Y, Katsuki M (2006). Decreased mutant frequency in embryonic brain of DNA polymerase beta null mice.. Mutagenesis.

[34] Fotiadou P, Henegariu O, Sweasy JB (2004). DNA polymerase β interacts with TRF2 and induces telomere dysfunction in a murine mammary cell line.. Cancer Research.

[35] McNees CJ, Conlan LA, Tenis N, Heierhorst J (2005). ASCIZ regulates lesion-specific Rad51 focus formation and apoptosis after methylating DNA damage.. Embo J.

[36] Oka H, Sakai W, Sonoda E, Nakamura J, Asagoshi K (2008). DNA damage response protein ASCIZ links base excision repair with immunoglobulin gene conversion.. Biochem Biophys Res Commun.

[37] Gembka A, Toueille M, Smirnova E, Poltz R, Ferrari E (2007). The checkpoint clamp, Rad9-Rad1-Hus1 complex, preferentially stimulates the activity of apurinic/apyrimidinic endonuclease 1 and DNA polymerase beta in long patch base excision repair.. Nucleic Acids Res.

[38] Stelzl U, Worm U, Lalowski M, Haenig C, Brembeck FH (2005). A human protein-protein interaction network: a resource for annotating the proteome.. Cell.

[39] Wang L, Bhattacharyya N, Chelsea DM, Escobar PF, Banerjee S (2004). A novel nuclear protein, MGC5306 interacts with DNA polymerase beta and has a potential role in cellular phenotype.. Cancer Res.

[40] Verdun RE, Karlseder J (2006). The DNA damage machinery and homologous recombination pathway act consecutively to protect human telomeres.. Cell.

[41] Hasan S, El-Andaloussi N, Hardeland U, Hassa PO, Burki C (2002). Acetylation regulates the DNA end-trimming activity of DNA polymerase β.. Molecular Cell.

[42] El-Andaloussi N, Valovka T, Toueille M, Hassa PO, Gehrig P (2007). Methylation of DNA polymerase beta by protein arginine methyltransferase 1 regulates its binding to proliferating cell nuclear antigen.. Faseb J.

[43] El-Andaloussi N, Valovka T, Toueille M, Steinacher R, Focke F (2006). Arginine methylation regulates DNA polymerase β.. Molecular Cell.

[44] Sobol RW (2008). CHIPping Away at Base Excision Repair.. Molecular Cell.

[45] Parsons JL, Tait PS, Finch D, Dianova, Allinson SL (2008). CHIP-Mediated Degradation and DNA Damage-Dependent Stabilization Regulate Base Excision Repair Proteins.. Mol Cell.

[46] Sobol RW, Foley JF, Nyska A, Davidson MG, Wilson SH (2003). Regulated over-expression of DNA polymerase β mediates early onset cataract in mice.. DNA Repair.

[47] Feng YQ, Lorincz MC, Fiering S, Greally JM, Bouhassira EE (2001). Position effects are influenced by the orientation of a transgene with respect to flanking chromatin.. Mol Cell Biol.

[48] Gao Q, Reynolds GE, Innes L, Pedram M, Jones E (2007). Telomeric transgenes are silenced in adult mouse tissues and embryo fibroblasts but are expressed in embryonic stem cells.. Stem Cells.

[49] Mehta AK, Majumdar SS, Alam P, Gulati N, Brahmachari V (2009). Epigenetic regulation of cytomegalovirus major immediate-early promoter activity in transgenic mice.. Gene.

[50] Maronpot RR (1999). Pathology of the Mouse: Reference and Atlas..

[51] Glassner BJ, Rasmussen LJ, Najarian MT, Posnick LM, Samson LD (1998). Generation of a strong mutator phenotype in yeast by imbalanced base excision repair.. Proceedings of the National Academy of Science.

[52] Hofseth LJ, Khan MA, Ambrose M, Nikolayeva O, Xu-Welliver M (2003). The adaptive imbalance in base excision-repair enzymes generates microsatellite instability in chronic inflammation.. Journal of Clinical Investigation.

[53] Kruman, Schwartz E, Kruman Y, Cutler RG, Zhu X (2004). Suppression of uracil-DNA glycosylase induces neuronal apoptosis.. J Biol Chem.

[54] Rinne M, Caldwell D, Kelley MR (2004). Transient adenoviral N-methylpurine DNA glycosylase overexpression imparts chemotherapeutic sensitivity to human breast cancer cells.. Molecular Cancer Therapeutics.

[55] Wang L, Bhattacharyya N, Rabi T, Wang L, Banerjee S (2007). Mammary carcinogenesis in transgenic mice expressing a dominant-negative mutant of DNA polymerase beta in their mammary glands.. Carcinogenesis.

[56] Cabelof DC, Ikeno Y, Nyska A, Busuttil RA, Anyangwe N (2006). Haploinsufficiency in DNA polymerase beta increases cancer risk with age and alters mortality rate.. Cancer Res.

[57] Bhattacharyya N, Banerjee S (1997). A variant of DNA polymerase β acts as a dominant negative mutant.. Proceedings of the National Academy of Science.

[58] Bu D, Cler LR, Lewis CM, Euhus DM (2004). A variant of DNA polymerase beta is not cancer specific.. J Invest Surg.

[59] Bergoglio V, Frechet M, Philippe M, Bieth A, Mercier P (2004). Evidence of finely tuned expression of DNA polymerase beta in vivo using transgenic mice.. FEBS Lett.

[60] Ward JM, Anver MR, Mahler JF, Devor-Henneman DE, Ward JM, Mahler JF, Maronpot RR, Sundberg JP (2000). Pathology of mice commonly used in genetic engineering (C57BL/6; 129; B6 129; and FVB/N).. Pathology of genetically engineered mice..

[61] Frith CH, Highman B, Burger G, Sheldon WD (1983). Spontaneous lesions in virgin and retired breeder BALB/c and C57BL/6 mice.. Lab Anim Sci.

[62] Erianne GS, Wajchman J, Yauch R, Tsiagbe VK, Kim BS (2000). B cell lymphomas of C57L/J mice; the role of natural killer cells and T helper cells in lymphoma development and growth.. Leuk Res.

[63] Rowlatt C, Franks LM, Sheriff MU, Chesterman FC (1969). Naturally occurring tumors and other lesions of the digestive tract in untreated C57BL mice.. J Natl Cancer Inst.

[64] Ward JM, Weisburger EK (1975). Intestinal tumors in mice treated with a single injection of N-nitroso-N-butylurea.. Cancer Res.

[65] Mizutani T, Yamamoto T, Ozaki A, Oowada T, Mitsuoka T (1984). Spontaneous polyposis in the small intestine of germ-free and conventionalized BALB/c mice.. Cancer Lett.

[66] Kimura S, Kawabe M, Ward JM, Morishima H, Kadlubar FF (1999). CYP1A2 is not the primary enzyme responsible for 4-aminobiphenyl-induced hepatocarcinogenesis in mice.. Carcinogenesis.

[67] Fernandez-Salguero PM, Ward JM, Sundberg JP, Gonzalez FJ (1997). Lesions of aryl-hydrocarbon receptor-deficient mice.. Vet Pathol.

[68] Takaku K, Miyoshi H, Matsunaga A, Oshima M, Sasaki N (1999). Gastric and duodenal polyps in Smad4 (Dpc4) knockout mice.. Cancer Res.

[69] Boivin GP, Molina JR, Ormsby I, Stemmermann G, Doetschman T (1996). Gastric lesions in transforming growth factor beta-1 heterozygous mice.. Lab Invest.

[70] Mahler M, Rozell B, Mahler JF, Merlino G, Devor-Henneman D, Ward JM, Mahler JF, Maronpot RR, Sundberg JP (2000). Pathology of the Gastrointestinal Tract of Genetically Engineered and Spontaneous Mutant Mice.. Pathology of genetically engineered mice..

[71] Ho SB, Lyftogt CT, Shekels LL, Niehans GA (1995). Experimental model of upper intestinal adenocarcinoma induced by N-methyl-N'-nitro-N-nitrosoguanidine in C57BL/6 mice.. Cancer Lett.

[72] Fischer HP, Zhou H (2003). [Pathogenesis and histomorphology of ampullary carcinomas and their precursor lesions. Review and individual findings].. Pathologe.

[73] Kimura W, Futakawa N, Zhao B (2004). Neoplastic diseases of the papilla of Vater.. J Hepatobiliary Pancreat Surg.

[74] Jean M, Dua K (2003). Tumors of the ampulla of Vater.. Curr Gastroenterol Rep.

[75] Esposito I, Friess H, Buchler MW (2001). Carcinogenesis of cancer of the papilla and ampulla: pathophysiological facts and molecular biological mechanisms.. Langenbecks Arch Surg.

[76] Yang K, Edelmann W, Fan K, Lau K, Kolli VR (1997). A mouse model of human familial adenomatous polyposis.. J Exp Zool.

[77] Jaiswal AS, Narayan S (2008). A novel function of adenomatous polyposis coli (APC) in regulating DNA repair.. Cancer Lett.

[78] Cockman-Thomas RA, Dunn DG, Innskeep W, Mondy WL, Swearengen JR (1994). Spontaneous osteosarcoma in a C57BL/6J mouse.. Lab Anim Sci.

[79] Madi K, De Paola D, Duarte F, Takyia C, Lima RJ (1990). Spontaneous amyloidosis in mice with malignant neoplasms.. Exp Pathol.

[80] Ruben Z, Rohrbacher E, Miller JE (1986). Spontaneous osteogenic sarcoma in the rat.. J Comp Pathol.

[81] Luz A, Gossner W, Murray AB, Jones TC, Mohr U, Hunt RD (1991). Osteosarcoma, Spontaneous and Radiation-Induced, Mouse.. Monographs on Pathology of Laboratory Animals Sponsored by the International Life Sciences Institute Cardiovascular and Musculoskeletal Systems..

[82] Wadsworth PF (1998). Tumors of the bone in C57BL/10J mice.. Lab Animal.

[83] Boorman GA, Montgomery CA, MacKenzie WF (1990). Pathology of the Fisher Rat..

[84] Wang L, Patel U, Ghosh L, Banerjee S (1992). DNA polymerase β mutations in human colorectal cancer.. Cancer Research.

[85] Grossblatt N (1996). Guide for the Care and Use of Laboratory Animals..

[86] NIEHS

[87] Patel AC, Anna CH, Foley JF, Stockton PS, Tyson FL (2000). Hypermethylation of the p16 (Ink4a) promoter in B6C3F1 mouse primary lung adenocarcinomas and mouse lung cell lines.. Carcinogenesis.

[88] Denkert C, Koch I, von Keyserlingk N, Noske A, Niesporek S (2006). Expression of the ELAV-like protein HuR in human colon cancer: association with tumor stage and cyclooxygenase-2.. Mod Pathol.

